# ID 190 - Carga da Doença e Estimativa de Produtividade Perdida Associada a Doenças Cardiovasculares na América do Sul

**DOI:** 10.5327/2237-9622.2025.v34s1.190

**Published:** 2025-11-25

**Authors:** Tayna Felicissimo Gomes de Souza Bandeira, Gabriela Bittencourt González Mosegui, Cid Manso de Mello Vianna, Fernando Antoñanzas, Alfonso Jesús Gil Lopez

**Keywords:** perda de produtividade, carga da doença, doenças cardiovasculares

## Abstract

**Introdução:**

As doenças cardiovasculares (DCV) são uma das principais causas de morbidade e mortalidade em todo o mundo, sendo responsáveis por 17,9 milhões de mortes em 2019. A carga dessas doenças na América Latina e no Caribe (ALC), especialmente na América do Sul (AS), tem aumentado ao longo dos anos, impulsionada por mudanças epidemiológicas, demográficas e de estilo de vida: seu impacto na saúde e na economia é significativo. Na AS, a perda de produtividade relacionada a essas doenças ainda não foi bem explorada. Uma análise dessas perdas proporcionaria uma compreensão adicional para o reconhecimento das prioridades de saúde e a tomada de decisões sobre a prevenção, o diagnóstico e o tratamento dessas doenças. O objetivo desse estudo foi descrever a carga da doença e estimar o impacto das perdas permanentes de produtividade causadas pelas DCV nos países sul-americanos.

**Método:**

Este é um estudo exploratório, de base populacional. Foi utilizada uma proxy método da Abordagem do Capital Humano (ACH) para estimar o custo das perdas permanentes de produtividade associadas às DCV. Para calcular este custo, somou-se os anos de vida produtiva perdidos (APLL) para cada morte, multiplicados pela proporção na força de trabalho (PFT) e pela taxa de emprego (TE), e, em seguida, pelo salário-mínimo anual ou paridade do poder de compra (PPC) em dólares americanos (US$) para cada país nos grupos etários economicamente ativos. Foram realizados cálculos separados para homens e mulheres. As medidas epidemiológicas por grupo etário padronizado entre os 12 países foram obtidas por meio do site do Institute for Health Metrics and Evaluation (IHME), que utiliza a avaliação do Estudo Global de Carga de Doenças, Lesões e Fatores de Risco (GBD) 2019. Análises de correlação foram realizadas utilizando indicadores econômicos, sociodemográficos e de saúde.

**Resultados:**

O número total de mortes por DCV em 2019 foi de quase 172 mil e os anos de vida produtiva perdidos foram de quase 2 milhões. O custo total das perdas permanentes de produtividade foi de aproximadamente US$ 3,7 bilhões com base no salário-mínimo anual e US$ 8 bilhões em paridade do poder de compra (PPC), representando 0,11% do produto interno bruto (PIB) da região. O custo por morte foi de US$ 23.000. O custo das perdas de produtividade variou entre os países e por sexo. As perdas de trabalho causadas por mortes precoces foram estimadas utilizando diferentes taxas de desconto. Há uma correlação entre indicadores de saúde sociodemográficos e econômicos e as taxas padronizadas de incidência e Anos de Vida Ajustados por Incapacidade (AVAI) nas doenças cardiovasculares.

**Conclusão:**

Este grupo de doenças impõe uma carga econômica significativa na América do Sul em termos de saúde e produtividade. A caracterização dos custos econômicos dessas doenças pode auxiliar os governos na alocação de recursos para desenvolver políticas e intervenções que reduzam essa carga

